# The efficacy and safety of four different PD-1 checkpoint inhibitors in advanced non-small cell lung cancer: a real-world retrospective study

**DOI:** 10.3389/fonc.2026.1833634

**Published:** 2026-04-28

**Authors:** Hanqi Wang, Zhiwei Xing, Xueru Ren, Rubing Bai, Yuxia Zou, Yuenan Wang, Huanhuan Zhang, Zhihong Zhang

**Affiliations:** 1 Department of Respiratory and Critical Care Medicine, The Second Affiliated Hospital of Soochow University, Suzhou, Jiangsu, China; 2 Department of Respiratory Oncology, The First Affiliated Hospital of University of Science and Technology of China (USTC) West District, Hefei, Anhui, China; 3 Department of Oncology, The Second Affiliated Hospital of Anhui Medical University, Hefei, Anhui, China; 4 Department of Respiratory and Critical Care Medicine, Bengbu First People’s Hospital, Bengbu, Anhui, China; 5 Department of Rheumatology, The people’s hospital of Xuancheng City, Xuancheng, Anhui, China; 6 Department of Respiratory Oncology, Lixin country People’s Hospital, Lixin, Bozhou, China; 7 Department of Respiratory Oncology, The sixth people’s hospital of Fuyang City, Fuyang, Anhui, China

**Keywords:** Efficacy, non-small cell lung cancer (NSCLC), PD-1 checkpoint inhibitors, real-world study, safety

## Abstract

**Background:**

While various PD-1 inhibitors are approved for advanced non-small cell lung cancer (NSCLC), direct comparisons of their real-world efficacy and safety are limited. This retrospective study evaluates four PD-1 inhibitors (pembrolizumab, sintilimab, tislelizumab, and camrelizumab) in real-world clinical practice to supplement registered clinical trial (RCT) data.

**Methods:**

We retrospectively screened 563 patients with stage IIIB-IV NSCLC who received pembrolizumab, sintilimab, tislelizumab, or camrelizumab (as monotherapy or combined with chemotherapy) between February 1, 2019, and December 31, 2022. The follow-up cutoff date was September 15, 2023.

**Results:**

A total of 409 eligible patients were included (pembrolizumab: 136; sintilimab: 115; tislelizumab: 123; camrelizumab: 35). For squamous NSCLC, no significant differences in effectiveness were observed among the four inhibitors, regardless of the treatment line. For nonsquamous NSCLC, second or later-line camrelizumab was associated with a higher risk of disease progression (progression-free survival [PFS] hazard ratio [HR] 2.29; 95% CI 1.01-5.19) and mortality (overall survival [OS] HR 3.14; 95% CI 1.28-7.74) compared to pembrolizumab, despite similar objective response rates. Regarding safety, immune-related adverse events (irAEs) were consistent and manageable. Notably, sintilimab demonstrated a significantly lower incidence of irAEs than the other three inhibitors across any grade (*P* = 0.004) and grades 3-5 (*P* = 0.005).

**Conclusions:**

In this real-world, single-center retrospective study, sintilimab and tislelizumab showed efficacy estimates broadly similar to pembrolizumab in advanced NSCLC within prespecified strata. Camrelizumab was associated with shorter PFS/OS in previously treated nonsquamous NSCLC; however, this finding is hypothesis-generating given small subgroup sizes, treatment heterogeneity, and potential residual confounding. Overall irAEs were manageable; differences in recorded incidence should be interpreted cautiously.

## Highlights

- In this single-center retrospective cohort, no statistically significant differences in efficacy were observed between pembrolizumab, sintilimab, and tislelizumab within prespecified strata.
- Camrelizumab showed numerically shorter PFS/OS in the second or later-line treated nonsquamous NSCLC; estimates were imprecise due to small sample size and potential residual confounding.
- Lower rates of recorded irAEs were observed with sintilimab; interpretation is limited by retrospective AE ascertainment and counting rules.

## 1 Introduction

Lung cancer is one of the most common malignancies. The annual morbidity and mortality rates are continue to rise, and patients are often diagnosed at advanced stages, which is the reason for the poor progression as treatment options are limited (1). Non-small cell lung cancer (NSCLC), as the most common histological type, accounts for more than 85% of all lung cancers (2). For NSCLC, early clinical treatments usually include surgery, radiotherapy, chemotherapy, molecular targeted therapy or combination therapy depending on the stage, histology, genetic alterations and patient condition. In the past decade, immunotherapy has also been recognized as an effective treatment for lung cancer due to the identification of molecular mechanisms underlying immune evasion by tumor cells against T cells (3–5). In 2015, U.S. The Food and Drug Administration approved pembrolizumab for the treatment of patients with advanced NSCLC (6). Subsequently, immunotherapy was officially introduced into China with the approval of pembrolizumab by China’s State Administration for the Supervision of Medicinal Products in March 2019 (7). The other three PD-1 inhibitors, sintilimab, tislelizumab, and camrelizumab, have been approved in China. However, most of the current clinical studies chose patients on conventional chemotherapy as the control group. Regrettably, this led to limited data for direct efficacy comparisons among the different PD-1 inhibitors. Furthermore, the current studies on the KEYNOTE 407 and KEYNOTE 189 have only evaluated the efficacy of first-line use of pembrolizumab in combination with chemotherapy in patients with lung cancer, and there have been no reports on the efficacy of second or later-line use of pembrolizumab in combination with chemotherapy (8, 9). Similarly, there is a lack of such reports in the series of 3 domestic PD-1 inhibitors in non-small cell lung cancer (Camel study, Rational study, Orient study) (10–15). In conclusion, the lack of a comprehensive assessment of the efficacy and safety of various PD-1 inhibitors has become a problem that clinicians need to address.

Therefore, a real-world retrospective observational study was conducted to explore potential risk factors that might affect the efficacy of PD-1 inhibitors, and then we explored the efficacy and safety of four types of PD-1 inhibitors based on different lines of treatments and various histologic features.

## 2 Materials and methods

### 2.1 Study design and patient characteristics

**Figure 1:**
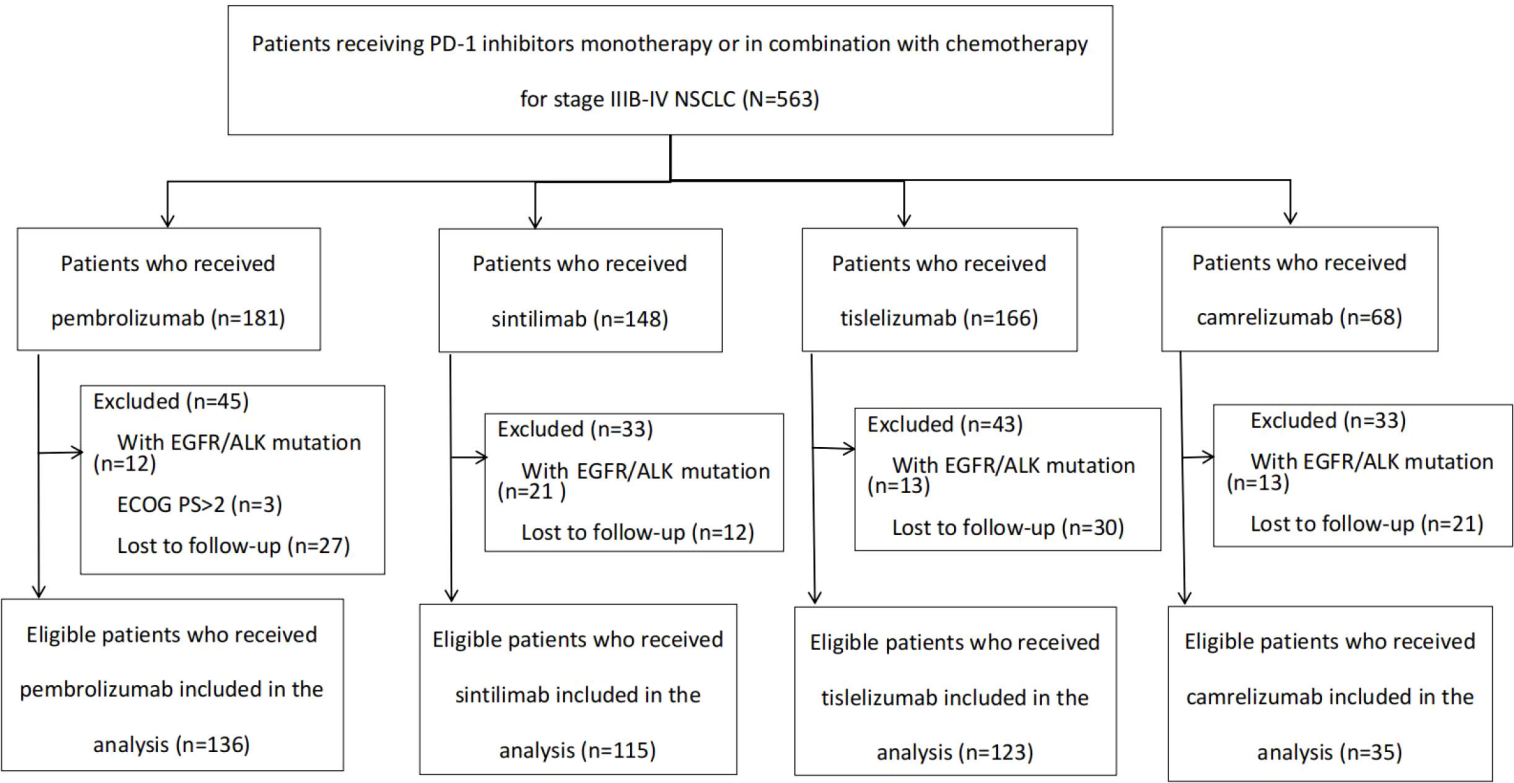
Flow chart of study profile.

563 patients with IIIB-IV NSCLC who received pembrolizumab, sintilimab, tislelizumab or camrelizumab monotherapy or in combination with chemotherapy at The First Affiliated Hospital of USTC from January 2019 through December 2022 were included for further screening, and the screening criteria for the study were as follows: (1) Patients with histologically or cytologically confirmed primary NSCLC; (2) age ≥18 years; (3) clinical stage IIIB–IV based on the 8th edition of the American Joint Committee on Cancer Tumor, Node, Metastasis (AJCC TNM) staging system; (4) EGFR and ALK driver gene-negative; (5) Eastern Cooperative Oncology Group (ECOG) performance status 0-2; (6) had measurable disease as per RECIST v1.1; (7) exclude cases where follow-up information was not available. As of September 15, 2023, a total of 409 patients met these criteria and were ultimately included in this analysis, and the screening process and results are shown in **Figure 1**. The characteristics of these patients included sex, age, smoking status, disease stage, EGOG performance status, PD-L1 expression, the presence of brain, liver or bone metastases and thoracic radiotherapy. PD-L1 expression was assessed by the tumor proportion score (TPS) and reported by using a three-point cut-off system: TPS<1%, TPS1%-49%, and TPS≥50%.

### 2.2 Treatment regimen

Patients received pembrolizumab, sintilimab, tislelizumab or camrelizumab (200 mg) intravenously every 21 days. Pembrolizumab, sintilimab, tislelizumab or camrelizumab treatment continued for 35 treatment cycles/2 years until disease progression, unacceptable toxicity, or the patient was lost to follow-up. The chemotherapy agents for patients with nonsquamous NSCLC included pemetrexed plus platinum with or without bevacizumab. The chemotherapy agents for patients with squamous NSCLC included docetaxel, paclitaxel, nab-paclitaxel, or gemcitabine with or without platinum. Chemotherapy agents were administered as follows: pemetrexed 500 mg/m^2^, intravenously; bevacizumab 15 mg/kg, intravenously; docetaxel 75 mg/m^2^, intravenously; paclitaxel 175 mg/m^2^, intravenously; nab-paclitaxel 100 mg/m^2^, intravenously; and gemcitabine 1,250 mg/m^2^, intravenously.

### 2.3 Assessments and endpoints

We selected progression-free survival (PFS), overall survival (OS) and objective response rate (ORR) as the primary endpoints, assessed as per RECIST v1.1. Secondary endpoints were assessed for, disease control rate (DCR) and irAEs. Objective tumor responses include response complete response (CR), partial response (PR), stable disease (SD), and progressive disease (PD). The ORR was defined as the CR plus PR rate. The DCR was defined as the combination of the CR, PR, and SD rates. The time from the start of treatment with PD-1 inhibitors until PD, death, or the end of the follow-up period was used to compute PFS. OS was defined by the time from receiving PD-1 inhibitors until death from any cause or the last follow-up. Radiologic assessments were performed per routine clinical practice; assessment intervals were not prespecified. For this retrospective analysis, progression and best overall response were abstracted from radiology reports and clinician documentation, and RECIST v1.1 was applied when sufficient imaging measurements were available. Adverse effects were evaluated based on the fourth version of the Common Terminology Criteria for Adverse Events (CTCAE).

### 2.4 Statistical analysis

Continuous variables were expressed as the mean (median), and comparisons among groups were made using one-way ANOVA. Categorical variables were summarized by the number (percentage) of patients, and comparisons among groups were made using the chi-square test. In addition, Pearson’s chi-square test and Fisher’s exact test were used to analyze differences in the incidence rate of irAEs, DCR, and ORR among various PD-1 inhibitors. Survival curves were generated using the Kaplan–Meier method. The Cox regression method was used for survival analyses. The Cox proportional hazards model was used for multivariable survival analysis. Hazard ratios (HRs) and 95% confidence intervals (CIs) were estimated using Cox proportional hazards models. The proportional hazards assumption was assessed using Schoenfeld residuals. Variables with P <0.10 in univariable analysis were considered for multivariable modeling; statistical significance was defined as two-sided P <0.05. < IBM SPSS Statistics version 27.0.

## 3 Results

### 3.1 Baseline characteristics of the 409 patients

A total of 409 patients with NSCLC who received PD-1inhibitors were included in our study. Their characteristics are given in **Table 1**.The median age was 66 years, males accounted for 345 cases (84.4%) and about 325(79.5%) patients had a history of smoking. The Eastern Cooperative Oncology Group Performance Status (ECOG-PS) was 0 or 1 in 381 patients (93.2%), and approximately 291(71.1%) patients treated with PD-1 inhibitors were at stage IV. Most patients(76.8%)did not have a PD-L1 testing, Among patients who had the PD-L1 testing, 26 had PD-L1 TPS ≤ 1%, 31 had PD-L1 TPS 1-49%, and 38 had PD-L1 TPS≥50%. 279 (68.2%) patients were treated with first-line therapy, 212 (51.8%) had distant metastases from the tumor, and 95(23.2%) were received thoracic radiotherapy (**Table 1**).

**Table 1:** Baseline clinical features of the 409 patients with NSCLC.

Characteristic	Number	Percentage
Median age(range),years	66(36-99)	100%
Age, years		
≤65	193	47.2%
>65	216	52.8%
Gender		
Male	345	84.4%
Female	64	15.7%
Smoking status		
Never	84	20.5%
Former/current	325	79.5%
Stage		
IIIB-IIIC	118	28.9%
IV	291	71.1%
ECOG performance status		
0-1	381	93.2%
2	28	6.8%
PD-L1 TPS		
Unavailable	314	76.8%
≤1%	26	6.4%
1-49%	31	7.6%
≥50%	38	9.3%
Distant metastases		
No	197	48.2%
Yes	212	51.8%
Thoracic radiotherapy		
No	314	76.8%
Yes	95	23.2%
Lines of immunotherapy		
First-line	279	68.2%
Second or later-line	130	31.8%
PD-1 inhibitors		
pembrolizumab	136	33.3%
sintilimab	115	28.1%
tislelizumab	123	30.1%
camrelizumab	35	8.6%

ECOG, Eastern Cooperative Oncology Group; PD-1inhibitors, programmed cell death-1 inhibitors; PD-L1 TPS, programmed cell death-ligand 1 tumor proportion score.

### 3.2 Analysis of prognostic factors

Univariate Cox regression analysis suggested that patients with baseline nonsquamous NSCLC (P = 0.098), receiving PD-1 inhibitors as first-line treatment (*P* < 0.001), and those treated with pembrolizumab, sintilimab, or tislelizumab (*P*=0.005) had longer PFS. Age, gender and three factors that showed close association (*P*<0.1) with PFS on univariate analyses were selected for multivariate analysis. Subsequent multivariate Cox regression analysis revealed that baseline nonsquamous NSCLC patients showed a significantly prolonged PFS compared to that of squamous NSCLC patients (HR, 0.76; 95% CI, 0.58-0.98, *P*=0.035) and patients who received a PD-1 inhibitor as a first-line treatment had a significantly prolonged PFS (HR, 0.63, 95% CI: 0.49-0.80, *P*<0.001). Moreover, patients treated with camrelizumab exhibited a significantly shorter PFS than those in the other three groups (*P*=0.009). In summary, we think that histologic features, lines of immunotherapy and PD-1 inhibitors were the three factors that have the greatest impact on survival, as detailed in **Table 2**. To further explore whether there was a difference in the efficacy of the four PD-1 inhibitors, we categorized 409 patients into a first-line treatment group and a second or later-line treatment group, and further subdivided by squamous group and nonsquamous NSCLC group.

**Table 2:** Univariate Cox regression and Multivariate Cox regression of progression free survival of all patients.

Variables	PFS					
	Univariate Cox regression			Multivariate Cox regression		
	HR	95%CI	*P* value	HR	95%CI	*P* value
Age(≤65 vs >65)	0.94	0.76-1.18	0.607	0.93	0.74-1.18	0.558
Gender(Female vs Male)	1.05	0.78-1.41	0.769	0.95	0.69-1.32	0.777
Smoking status(Never vs Former,current)	0.99	0.76-1.30	0.963			
ECOG performance status (0–1 vs 2)	0.70	0.46-1.06	0.696			
Histologic features (Nonsquamous vs squamous)	0.83	0.66-1.04	0.098*	0.76	0.58-0.98	0.035*
Stage(IV vs IIIB-IIIC)	1.01	0.79-1.29	0.944			
Distant metastases(Yes vs No)	1.17	0.93-1.46	0.177			
Thoracic radiotherapy(Yes vs No)	0.98	0.76-1.27	0.881			
Lines of immunotherapy (First-line vs second or later-line)	0.66	0.53-0.84	<0.001	0.63	0.49-0.80	<0.001*
PD-1 inhibitors						
pembrolizumab	1.0(ref)		0.005*	1.0(ref)		0.009*
sintilimab	1.05	0.79-1.41	0.733	0.90	0.67-1.22	0.510
tislelizumab	1.31	0.99-1.74	0.059*	1.19	0.89-1.60	0.238
camrelizumab	1.96	1.31-2.93	<0.001*	1.78	1.18-2.67	0.006*

age,gender,histologic features,lines of immunotherapy and PD-1 inhibitors selected for multivariate analysis.

*Variables with P <0.10 in univariable analysis were considered for multivariable modeling; statistical significance was defined as two-sided P <0.05. PFS, progression free survival; HR, hazard ratio; 95% CI, 95% confidence interval; ECOG, Eastern Cooperative Oncology Group; PD-1inhibitors, programmed cell death-1 inhibitors.

### 3.3 Patients treated with PD-1 inhibitors as first-line treatment

#### 3.2.1 Patient characteristics

A total of 184 patients with squamous NSCLC using PD-1 inhibitors in the first-line setting were evaluated in the study. Of these patients, 55 were treated with pembrolizumab, 46 with sintilimab, 72 with tislelizumab, and 11 with camrelizumab. The median age at diagnosis for the entire cohort was 69 years (range: 43–84 years). When broken down by treatment groups, the median ages were as follows: 71 years for the pembrolizumab group, 68 years for both the sintilimab and camrelizumab groups, and 69 years for the tislelizumab group. Baseline clinical characteristics such as age, sex, smoking status and disease stage, ECOG performance status, PD-L1 expression, and the presence of brain, liver and bone metastases, thoracic radiotherapy history, as well as treatment strategy, showed no significant differences among the groups. The baseline demographic and clinical characteristics of the study group are shown in **Supplementary Table 1**. The baseline demographics and disease characteristics were well balanced among the groups.

Additionally, 95 patients with nonsquamous NSCLC were treated with PD-1 inhibitors as a first-line treatment. The treatment distribution was as follows: pembrolizumab for 48 patients, sintilimab for 19, tislelizumab for 23, and camrelizumab for 5 patients. Notably, the sintilimab group had a significantly lower proportion of patients with ECOG-PS scores of 0-1 (79.0%) than the pembrolizumab group (97.9%) (*Pb* = 0.008) (**Supplementary Table 2**). Despite this, other baseline characteristics remained balanced across the groups.

#### 3.3.2 Efficacy

**Figure 2:**
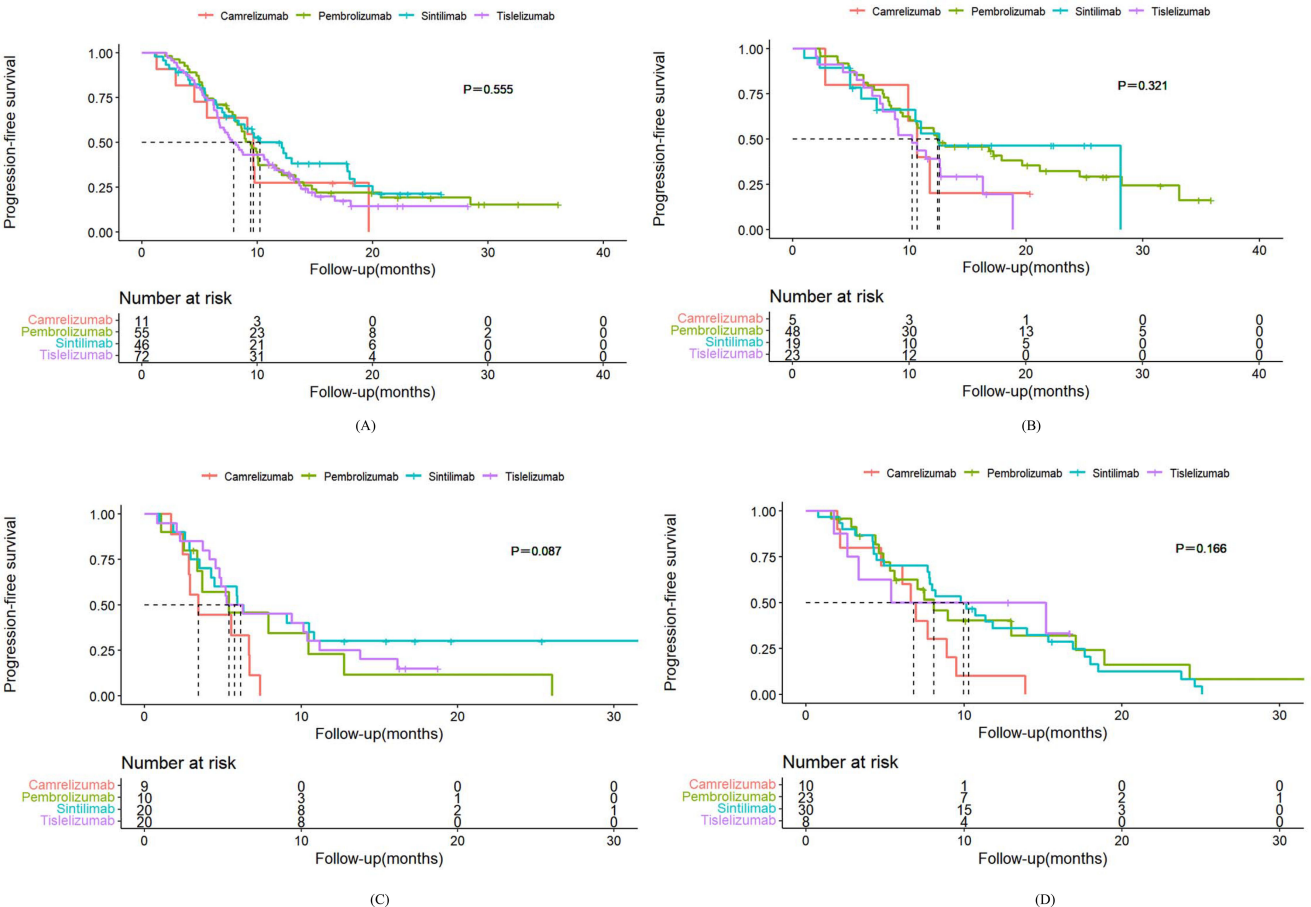
2Kaplan-Meier plot of PFS for different PD-1 inhibitors. **(A)** first-line therapy for squamous NSCLC. **(B)** first-line therapy for nonsquamous NSCLC. **(C)** second or later-line therapy for squamous NSCLC. **(D)** second or later-line therapy for nonsquamous NSCLC.

**Figure 3:**
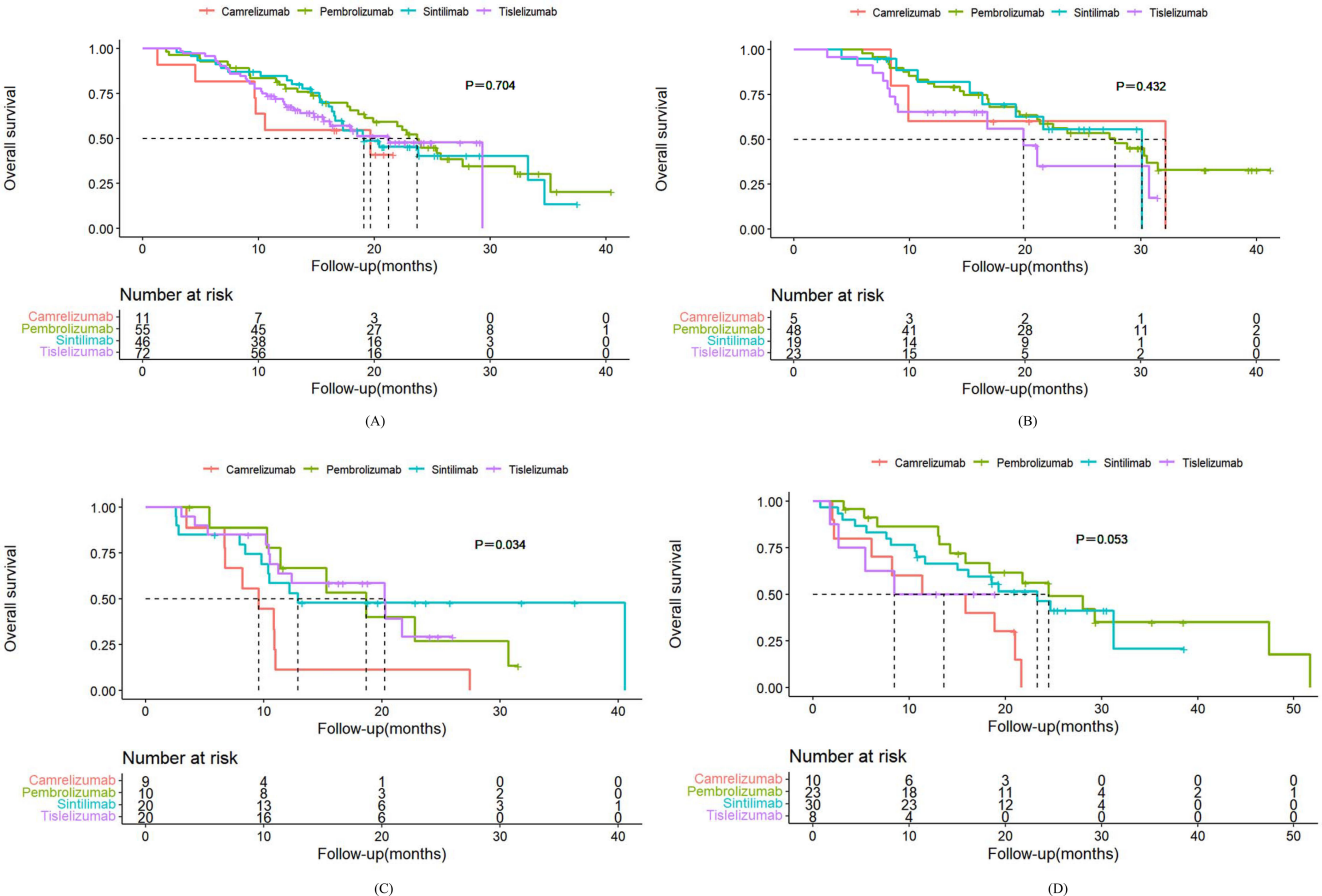
Kaplan-Meier plot of OS for different PD-1 inhibitors. **(A)** first-line therapy for squamous NSCLC. **(B)** first-line therapy for nonsquamous NSCLC. **(C)** second or later-line therapy for squamous NSCLC. **(D)** second or later-line therapy for nonsquamous NSCLC.

Among patients with squamous NSCLC receiving first-line treatment with PD-1 inhibitors, the median PFS was as follows: 9.4 months in the pembrolizumab group, 10.2 months in the sintilimab group, 7.8 months in the tislelizumab group, and 9.6 months in the camrelizumab group (**Figure 2A**). There was no statistically significant difference in PFS among the sintilimab group (HR, 0.86; 95% CI, 0.54-1.36), the tislelizumab group (HR, 1.16; 95% CI, 0.78-1.73) and the camrelizumab group (HR, 1.16; 95% CI, 0.57-2.39) when compared to the pembrolizumab group (**Table 3**). Additionally, the median OS for the pembrolizumab, sintilimab, tislelizumab, and camrelizumab groups was 23.7 months, 19.1 months, 21.2 months, and 19.6 months, respectively (**Figure 3A**). Similar to PFS, the variations in OS were not statistically significant among the sintilimab group (HR, 1.12; 95% CI, 0.66-1.90), the tislelizumab group (HR, 1.25; 95% CI, 0.75-2.07) and the camrelizumab group (HR, 1.60; 95% CI, 0.66-3.87) when compared to the pembrolizumab group (**Table 4**). The ORR was 58.4% versus 52.2% versus 58.3% versus 63.6% (*P*=0.869), and the DCR was 92.7% versus 91.3% versus 93.1% versus 90.9% (*P* = 0.963) in the pembrolizumab group, sintilimab group, tislelizumab group and camrelizumab group, respectively (**Supplementary Table 5**).

**Table 3:** Progression-free survival of patients and difference in efficacy between PD-1inhibitors.

	Cox regression			
Variables	No.of events	Median PFS, months(95% CI)	HR(95%CI)	*P* value
First-line				
Squamous NSCLC				
Pembrolizumab	55(42)	9.4(8.0-10.2)	1.0(ref)	
Sintilimab	46(30)	10.2(6.6-13.8)	0.86(0.54-1.36)	0.505
Tislelizumab	72(57)	7.8(6.2-9.4)	1.16(0.78-1.73)	0.456
Camrelizumab	11(9)	9.6(5.2-14.0)	1.16(0.57-2.39)	0.691
Nonsquamous NSCLC				
Pembrolizumab	48(34)	12.4(5.7-19.1)	1.0(ref)	
Sintilimab	19(10)	12.5(5.4-19.6)	0.96(0.47-1.96)	0.920
Tislelizumab	23(18)	10.2(8.1-12.3)	1.66(0.92-3.00)	0.094
Camrelizumab	5(4)	10.7(9.0-12.4)	1.51(0.53-4.30)	0.438
Second or later-line				
Squamous NSCLC				
Pembrolizumab	10(9)	5.4(0-11.4)	1.0(ref)	
Sintilimab	20(14)	5.9(3.3-8.5)	0.65(0.28-1.51)	0.316
Tislelizumab	20(17)	5.2(3.2-7.2)	0.82(0.36-1.87)	0.639
Camrelizumab	9(9)	3.4(1.9-4.9)	1.88(0.72-4.89)	0.194
Nonsquamous NSCLC				
Pembrolizumab	23(17)	8.1(5.6-10.6)	1.0(ref)	
Sintilimab	30(28)	9.8(6.2-13.4)	1.06(0.57-1.97)	0.827
Tislelizumab	8(5)	5.4(0-19.5)	1.01(0.36-2.82)	0.984
Camrelizumab	10(10)	6.6(5.2-8.0)	2.29(1.01-5.19)	0.046*

*The difference was statistically significant, with P <0.05. PFS, Progression-free survival; NSCLC, non-small cell lung cancer.

**Table 4:** Overall survival of patients and difference in efficacy between PD-1inhibitors.

	Cox regression			
Variables	No.of events	Medain OS, months(95% CI)	HR(95%CI)	*P* value
First-line				
Squamous NSCLC				
Pembrolizumab	55(32)	23.7(21.3-26.1)	1.0(ref)	
Sintilimab	46(25)	19.1(13.3-24.9)	1.12(0.66-1.90)	0.671
Tislelizumab	72(32)	21.2(17.9-24.5)	1.25(0.75-2.07)	0.395
Camrelizumab	11(6)	19.6(5.8-33.4)	1.60(0.66-3.87)	0.300
Nonsquamous NSCLC 0.470				
Pembrolizumab	48(27)	27.8(19.9-35.7)	1.0(ref)	
Sintilimab	19(8)	30.1	0.85(0.48-2.41)	0.852
Tislelizumab	23(12)	19.8(14.5-25.1)	1.74(0.87-3.49)	0.119
Camrelizumab	5(3)	32.1	1.30(0.39-4.29)	0.671
Second or later-line				
Squamous NSCLC				
Pembrolizumab	10(7)	18.6(9.4-27.8)	1.0(ref)	
Sintilimab	20(11)	12.9(1.3-24.5)	0.80(0.30-2.10)	0.648
Tislelizumab	20(11)	20.2(9.9-30.5)	0.90(0.34-2.36)	0.836
Camrelizumab	9(9)	9.5(5.7-12.3)	2.62(0.96-7.23)	0.061
Nonsquamous NSCLC				
Pembrolizumab	23(14)	24.5(14.0-35.0)	1.0(ref)	
Sintilimab	30(17)	23.3(15.3-31.1)	1.27(0.60-2.67)	0.536
Tislelizumab	8(4)	8.5	2.15(0.67-6.91)	0.199
Camrelizumab	10(9)	11.3(0-23.1)	3.14(1.28-7.74)	0.013*

*The difference was statistically significant, with P <0.05. OS,overall survival; NSCLC, non-small cell lung cancer.

For first-line treatments, patients with nonsquamous NSCLC receiving pembrolizumab, sintilimab, tislelizumab, or camrelizumab had median PFS of 12.4 months, 12.5 months, 10.2 months, and 10.7 months, respectively (**Figure 2B**). Their corresponding mOS values were 30.3 months, 38.9 months, 19.8 months, and 32.1 months (**Figure 3B**). The results of survival analysis showed that there was no significant difference in efficacy between the various PD-1 inhibitors in this subgroup. (**Table 3**, **4**). The ORR was 50.0% versus 57.9% versus 60.9% versus 60.0% (*P*=0.822), and the DCR was 89.6% versus 89.5% versus 91.3% versus 80.0% (*P* = 0.906) in the pembrolizumab group, sintilimab group, tislelizumab group and camrelizumab group, respectively (**Supplementary Table 6**).

### 3.4 Patients treated with PD-1 inhibitors as second or later-line treatment

#### 3.4.1 Patient characteristics

In this study, 59 patients with squamous NSCLC undergoing second or later-line PD-1 inhibitors were analyzed. This included 10 patients treated with pembrolizumab, 20 with sintilimab, 20 with tislelizumab, and 9 with camrelizumab. In the sintilimab group, a higher proportion of patients were over 65 years old (65.0%), whereas in the camrelizumab group, a greater percentage were 65 years old or younger (88.9%). This difference in age distribution between the two groups was statistically significant (*Pa* = 0.007) (**Supplementary Table 3**). Apart from this, there were no significant differences in baseline clinical characteristics among these four groups. Additionally, 71 nonsquamous NSCLC patients received PD-1 inhibitors as second or later-line treatment. The baseline clinical characteristics of the four groups were essentially consistent, with only the proportion of patients in stage IV being significantly higher in the sintilimab group than in the camrelizumab group (*Pc* = 0.002) (**Supplementary Table 4**).

#### 3.4.2 Efficacy

For squamous NSCLC patients receiving pembrolizumab, sintilimab, tislelizumab, or camrelizumab as second or later-line treatment, the median PFS(mPFS) was 5.4 months, 5.9 months, 5.2 months, and 3.4 months, respectively (**Figure 2C**). The corresponding median OS(mOS) values were 18.6 months, 12.9 months, 20.2 months, and 9.5 months (**Figure 3C**). There were also no statistically significant differences in PFS and OS among these groups (**Table 3**, **4**). The ORR was 40.0% in the pembrolizumab group, 35.0% in the sintilimab group, 30.0% in the tislelizumab group, and 33.3% in the camrelizumab group (*P* = 0.957). The DCR was 80.0% versus 80.0% versus 75.0% versus 66.7% (*P* = 0.872) (**Supplementary Table 7**).

In the nonsquamous NSCLC groups, the median PFS was as follows: pembrolizumab (8.1 months), sintilimab (9.8 months), tislelizumab (5.4 months), and camrelizumab (6.6 months) (**Figure 2D**). The corresponding mOS values were 24.5 months, 23.3 months, 8.5 months, and 11.3 months (**Figure 3D**). It is worth mentioning that we observed a higher risk of disease progression (HR, 2.29; 95% CI, 1.01-5.19) and a higher mortality risk (HR, 3.14; 95% CI, 1.28-7.74) in the camrelizumab group than in the pembrolizumab group (**Table 3**, **4**). The ORR was 43.5% versus 36.7% versus 37.5% versus 30.0% (*P* = 0.899), and the DCR was 87.0% versus 90.0% versus 87.5% versus 80.0% (*P* = 0.877) in the pembrolizumab group, sintilimab group, tislelizumab group and camrelizumab group, respectively (**Supplementary Table 8**).

### 3.5 Safety and toxicity of 409 patients

The immune-related adverse events (irAEs) are described in **Table 5**. irAEs occurred in 37.5% (grade 3 to 5, 22.8%) of patients in the pembrolizumab group, 18.3% (grade 3 to 5, 7.8%) of patients in the sintilimab group, 32.5% (grade 3 to 5, 19.5%) of patients in the tislelizumab group and 20% (grade 3 to 5, 8.6%) of patients in the camrelizumab group. We found that the toxicity of sintilimab was less than that of the other three PD1 inhibitors both at any grade (*P* = 0.004) and grade 3 to 5 (*P* = 0.005). The most common irAEs in the pembrolizumab group were hypothyroidism (10.3%) and rash (7%) in the sintilimab group, pneumonia (13.2%) in the tislelizumab group and reactive capillary endothelial proliferation (RCCEP) (11.4%) in the camrelizumab group. Immune-related hypophysitis occurred only in the pembrolizumab group, immune-related cystitis only in the sintilimab group, and immune-related RCCEP only in the camrelizumab group. The incidence of immune-related pneumonia was higher in the tislelizumab group than in the other three groups (*P* = 0.029). The most common grade 3 to 5 irAEs were pneumonia in both the pembrolizumab group and the tislelizumab group, hypothyroidism in the sintilimab group and RCCEP in the camrelizumab group. In addition, no deaths due to irAEs were observed in either group (**Table 5**).

**Table 5:** Incidence of immune-related adverse events among treated patients.

Variables	pembrolizumab (n=136)		sintilimab (n=115)		tislelizumab (n=123)		camrelizumab (n=35)		
Immune-related AEs, n (%)	Any Grade	Grade 3-5	Any Grade	Grade 3-5	Any Grade	Grade 3-5	Any Grade	Grade 3-5	*P* (any grade/grade 3 -5)
Any irAE	51(37.5)	31(22.8)	21(18.3)	9(7.8)	40(32.5)	24(19.5)	7(20.0)	3(8.6)	0.004*/0.005*
hematotoxicity	3(2.2)	3(2.2)	0	0	2(1.6)	2(1.6)	0	0	0.472/0.472
pneumonia	8(5.9)	6(4.4)	3(2.6)	1(0.9)	15(13.2)	8(6.5)	2(5.7)	1(2.9)	0.029*/0.156
enteritis	9(6.6)	0	1(0.9)	0	5(4.1)	4(3.1)	0	0	0.328/0.013*
hepatotoxicity	8(5.9)	5(3.7)	2(1.7)	1(0.9)	5(4.1)	2(1.6)	0	0	0.264/0.450
kidney damage	6(4.4)	2(1.5)	2(1.7)	1(0.9)	3(2.4)	1(0.8)	0	0	0.543/1.000
cystitis	0	0	1(0.9)	1(0.9)	0	0	0	0	0.367/0.367
hypophysitis	1(0.7)	1(0.7)	0	0	0	0	0	0	1.000/1.000
hypothyroidism	14(10.3)	5(3.7)	5(4.3)	3(2.6)	5(4.1)	2(1.6)	0	0	0.057/0.662
hyperthyroidism	2(1.5)	0	1(0.9)	1(0.9)	1(0.8)	0	0	0	1.000/0.367
hyperglycemia	2(1.5)	1(0.7)	1(0.9)	1(0.9)	0	0	0	0	0.730/0.800
cardiotoxicity	2(1.5)	2(1.5)	1(0.9)	0	6(4.9)	2(1.6)	2(5.7)	1(2.9)	0.127/0.380
rash	8(5.9)	3(2.2)	8(7.0)	2(1.7)	6(4.9)	3(2.4)	0	0	0.484/1.000
RCCEP	0	0	0	0	0	0	4(11.4)	2(5.7)	<0.001*/0.007*

Incidence of each irAE category is reported as the number (%) of patients experiencing ≥1 event of that category (patient-level). A patient could contribute to multiple categories if multiple irAE types occurred; therefore, category counts may sum to more than the number of patients with any irAE.

AEs,adverse events; RCCEP, reactive cutaneous capillary endothelial proliferation.

*The difference was statistically significant, with P <0.05.

## 4 Discussion

Immunotherapy has become an effective and viable alternative treatment for advanced NSCLC. However, due to cost constraints and limited indications for new drugs or therapeutic combinations, pembrolizumab has not yet been included in the current national health insurance reimbursement policy. This situation has led to a significant price difference among these four PD-1 inhibitors, with prices ranging from approximately 1,000-20,000 Chinese Yuan. Additionally, the clinical and sociological characteristics of the real-world oncology population are much more complex than those of patients participating in randomized clinical trials. Recent studies have explored the differences in efficacy and safety of different ICIs in the real world, but these studies lack a comparison of the efficacy and safety differences of various ICIs (16–18). To the best of our knowledge, this is the first time the efficacy and safety of pembrolizumab, sintilimab, tislelizumab and camrelizumab have been compared across different pathological types and treatment lines.

For squamous NSCLC patients receiving first-line treatment with PD-1 inhibitors, the clinical trials KETNOTE-407 (8), ORIENT-12 (11), Rationale 307 (13), and Camel-sq (14) have shown that the median PFS for pembrolizumab, sintilimab, tislelizumab, and camrelizumab ranges from 5.5 to 8.5 months, with an ORR between 44.7% and 72.5%. In our study, the ORR ranged from 52.2% to 63.6%, similar to these clinical trials, and no significant differences were observed in ORR and DCR among these four PD-1 inhibitors. Furthermore, although our study showed a longer median PFS for these PD-1 inhibitors (7.8-10.2 months) compared to that from RCTs, there was no significant difference in PFS among these groups. However, this extension may be attributed to a larger proportion of patients in our subgroup with locally advanced, unresectable, stage IIIB-IIIC patients and fewer patients with liver or brain metastases. Additionally, unlike the ORIENT-12 study, which used gemcitabine and platinum-based drugs, our study predominantly combined sintilimab with paclitaxel or nab-paclitaxel and platinum-based treatments, which have been shown to be more effective than gemcitabine (19).The median OS for these PD-1 inhibitors in this study ranging from 19.1-23.7 months, and there was also no significant difference in OS among these groups. In a prospective phase 2 study by Yi-Long Wu et al., directly comparing two anti-PD-1 antibodies as first-line treatment for NSCLC, it was found that sintilimab and pembrolizumab have similar efficacy in patients with advanced NSCLC, regardless of PD-1 expression levels (20).This result is also consistent with our study findings.

Regarding patients with nonsquamous NSCLC receiving first-line immunotherapy, clinical trials such as KETNOTE-189 (9), ORIENT-11 (10), Rationale 304 (12), and Camel (15) have demonstrated that pembrolizumab, sintilimab, tislelizumab, and camrelizumab have a median PFS ranging from 8.9 to 11.3 months and median OS(22.0-27.9 months), with ORR varying between 48.3% and 64.8%. In our study, these drugs exhibited a median PFS range of 10.2 to 12.5 months, a median OS range of 19.8-32.1 months and an ORR range of 50% to 60.9%, consistent with the results of these clinical trials. Furthermore, no significant differences in PFS, OS, ORR, or DCR were observed among the four groups.

Furthermore, as second-line and later treatments, data from KETNOTE-010 (21), ORIENT-3 (22), and Rationale-303 (23) indicate that monotherapy with PD-1 inhibitors is more effective than docetaxel. However, in real-world settings, most patients received PD-1 inhibitors combined with chemotherapy or targeted therapy as second- or later-line treatments after first-line chemotherapy failed. The current research lacked a cross-sectional comparison of the efficacy in patients using second-line pembrolizumab, sintilimab, tislelizumab, or camrelizumab. Therefore, in our study, based on histologic status, patients treated with these PD-1 inhibitors as second or later-line therapy were categorized into squamous NSCLC and nonsquamous NSCLC groups. In the squamous NSCLC group, pembrolizumab had a median PFS of 5.4 months and an ORR of 43.5%, while sintilimab showed a median PFS of 5.9 months and an ORR of 35.0%, closely consistent with Zhang et al.’s study, which reported a median PFS of 5.0 months and an ORR of 36.7% (24), and no significant difference was observed compared to pembrolizumab. Tislelizumab had a median PFS of 5.2 months and an ORR of 30.0%, which was also not significantly different from that of pembrolizumab. In Chen et al.’s study, the camrelizumab combined chemotherapy group had a median PFS of 5.0 months and an ORR of 35.85%. However, the camrelizumab group in our study exhibited a shorter median PFS of 3.4 months and an ORR of 33.3%, and this difference might be due to Chen et al. not excluding patients with adenocarcinoma (25). Although the median PFS was shorter in the camrelizumab group than in the pembrolizumab group, no statistically significant difference was observed (HR = 1.88, 95% CI, 0.72-4.89). In the nonsquamous NSCLC group, pembrolizumab showed a median PFS of 8.1 months and an ORR of 43.5%, which is roughly similar to the findings in the study by Oscar et al., which showed a median PFS of 9.5 months and an ORR of 42.5% for patients without EGFR/ALK mutations (26). In our study, sintilimab had a median PFS of 9.8 months and an ORR of 36.7%, showing no significant difference from pembrolizumab. Although the tislelizumab group in our study had a lower median PFS of 5.4 months and an ORR of 37.5% compared to the pembrolizumab group, these differences were not statistically significant. Notably, compared to the pembrolizumab group, the camrelizumab group exhibited a similar ORR but a shorter median PFS (6.6 months) and median OS(11.3 months), with a statistically significant difference in PFS (HR, 2.29; 95% CI, 1.01-5.19) and OS(HR,3.14;95% CI,1.28-7.74). However, given the wide confidence interval, it appears that the observed significant difference between the camrelizumab and pembrolizumab groups in our study could be attributed to the small sample size.

In terms of safety analysis, our study found that sintilimab had lower toxicity than the other three PD-1 inhibitors at any grade (*P* = 0.004) and at grades 3 to 5 (*P* = 0.005). Among all patients receiving immunotherapy, immune-related adverse events (irAEs) were generally safe and manageable, with most irAEs being grade 1-2, and no irAE-related deaths occurred. The three most common irAEs of pembrolizumab and sintilimab were hypothyroidism, rash, and pneumonia, consistent with Shi et al.’s report (7). Notably, the three most common irAEs for tislelizumab were pneumonia, rash, and cardiotoxicity. Myocarditis is one of the most fatal immune-related adverse reactions associated with immune checkpoint inhibitor therapy, with a mortality rate of up to 50% (27, 28). Fortunately, in our study, no patients died from severe myocarditis. RCCEP was exclusively observed in the camrelizumab group and was also the most frequent immune-related adverse event of this group, a finding consistent with the results of the Camel-sq study.

The present study has some limitations. First, it is a retrospective study in one center and the number of each group is small. Hence, information bias is unavoidable and future studies with larger sample sizes are needed to strengthen our conclusions. Second, only 95 (23.2%) patients had baseline PD-L1 expression information. We did not include PD-L1 expression in the analysis of prognostic factors. Third, PFS and response assessment in real-world retrospective settings are subject to measurement heterogeneity because imaging intervals, modality, and documentation are not standardized as in prospective trials. Differences in scan timing between treatment groups may introduce bias in comparative PFS estimates. Therefore, numerical comparisons of PFS/ORR with RCTs should be interpreted cautiously.

## 5 Conclusions

In conclusion, this single-center retrospective study provides real-world comparative data on four PD-1 inhibitors across histology and lines of therapy. No clear efficacy differences were identified between pembrolizumab, sintilimab, and tislelizumab within prespecified strata. An association between camrelizumab and shorter PFS/OS was observed in previously treated nonsquamous NSCLC, which requires confirmation in larger, preferably multicenter studies with robust confounding control. Safety profiles were broadly manageable; observed differences in recorded irAE rates should be interpreted cautiously.

## Data Availability

The raw data supporting the conclusions of this article will be made available by the authors, without undue reservation.
