# Supplementary material for: The efficacy and safety of four different PD-1 checkpoint inhibitors in advanced non-small cell lung cancer: a real-world retrospective study
Source: Front Oncol. 2026 Apr 28;16:1833634. doi: 10.3389/fonc.2026.1833634 (PMC13160789; doi:10.3389/fonc.2026.1833634)
Supplement: Supplementary file 1 [file Supplementaryfile1.docx]

Table S1. Demographic and clinical characteristics of the squamous NSCLC patients receiving first-line treatment with PD-1 inhibitors

| Characteristics | Total  (N=184) | pembrolizumab  (N=55) | sintilimab  (N=46) | tislelizumab  (N=72) | camrelizumab  (N=11) | *P* value |
| --- | --- | --- | --- | --- | --- | --- |
| Median age(range),years | 69(43-84) | 71(43-84) | 69(51-82) | 68(50-82) | 68(54-77) | 0.501 |
| Age,years |  |  |  |  |  |  |
| ≤65 | 58(31.5) | 14(25.5) | 14(30.4) | 28(38.9) | 2(18.2) | 0.298 |
| >65 | 126(68.5) | 41(74.5) | 32(69.6) | 44(61.1) | 9(81.8) |  |
| Gender |  |  |  |  |  |  |
| Male | 174(94.6) | 52(94.6) | 44(95.7) | 67(94.6) | 11(100) | 0.787 |
| Female | 10(5.4) | 3(5.4) | 2(4.4) | 5(6.9) | 0 |  |
| Smoking status, n (%) |  |  |  |  |  | 0.418 |
| Never | 21(11.4) | 4(7.3) | 4(8.7) | 11(15.3) | 2(18.2) |  |
| Former/current | 163(88.6) | 51(92.7) | 42(91.3) | 61(84.7) | 9(81.8) |  |
| Stage,n(%) |  |  |  |  |  | 0.565 |
| IIIB-IIIC | 80(43.5) | 25(45.5) | 22(47.8) | 27(37.5) | 6(54.6) |  |
| IV | 104(56.5) | 30(54.6) | 24(52.2) | 45(62.5) | 5(45.5) |  |
| ECOG performance status,n(%) |  |  |  |  |  | 0.638 |
| 0-1 | 176(95.7) | 54(98.2) | 44(95.7) | 68(94.4) | 10(90.9) |  |
| 2 | 8(4.4) | 1(1.8) | 2(4.4) | 4(5.6) | 1(9.1) |  |
| PD-L1 TPS, n(%) |  |  |  |  |  | 0.644 |
| Unavailable | 142(77.2) | 42(76.4) | 37(80.4) | 54(75.0) | 9(81.8) |  |
| ≤1 % | 12(6.5) | 3(5.5) | 2(4.4) | 5(6.9) | 2(18.2) |  |
| 1-49% | 15(8.2) | 5(9.1) | 2(4.4) | 8(11.1) | 0 |  |
| ≥50% | 15(8.2) | 5(9.1) | 5(10.9) | 5(6.9) | 0 |  |
| Brain metastases, n (%) |  |  |  |  |  | 0.151 |
| No | 170(92.4) | 52(94.6) | 45(97.8) | 64(88.9) | 9(81.8) |  |
| Yes | 14(7.6) | 3(5.4) | 1(2.2) | 8(11.1) | 2(18.2) |  |
| Liver metastases, n(%) |  |  |  |  |  | 0.447 |
| No | 169(91.9) | 51(92.7) | 44(95.7) | 65(90.3) | 9(81.8) |  |
| Yes | 15(8.1) | 4(7.3) | 2(4.3) | 7(9.7) | 2(18.2) |  |
| Bone metastases, n(%) |  |  |  |  |  | 0.441 |
| No | 134(72.8) | 43(78.2) | 33(71.7) | 52(72.2) | 6(54.6) |  |
| Yes | 50(27.2) | 12(21.8) | 13(28.3) | 20(27.8) | 5(45.4) |  |
| Thoracic radiotherapy, n(%) |  |  |  |  |  | 0.396 |
| No | 142(77.2) | 42(76.4) | 38(82.6) | 52(72.2) | 10(90.9) |  |
| Yes | 42(22.8) | 13(23.6) | 8(17.4） | 20(27.8) | 1(9.1) |  |
| Treatment strategy, n(%) |  |  |  |  |  | 0.151 |
| Monotherapy | 10(5.4) | 1(1.8) | 2(4.3) | 5(6.9) | 2(18.2) |  |
| Combination with chemotherapy | 174(94.6) | 54(98.2) | 44(95.7) | 67(93.1) | 9(81.8) |  |

Abbreviations: ECOG, Eastern Cooperative Oncology Group; PD-1inhibitors, programmed cell death-1 inhibitors; PD-L1 TPS, programmed cell death-ligand 1 tumour proportion score.

Table S2. Demographic and clinical characteristics of the nonsquamous NSCLC patients receiving first-line treatment with PD-1 inhibitors

| Characteristics | Total  (N=95) | pembrolizumab  (N=48) | sintilimab  (N=19) | tislelizumab  (N=23) | camrelizumab  (N=5) | *P* value |
| --- | --- | --- | --- | --- | --- | --- |
| Median age(range),years | 63(36-99) | 65(36-99) | 61(39-79) | 62(52-78) | 68(56-85) | 0.430 |
| Age,years |  |  |  |  |  | 0.327 |
| ≤65 | 56(59.0) | 25(52.1) | 13(68.4) | 16(69.6) | 2(40.0) |  |
| ＞65 | 39(41.0) | 23(47.9) | 6(31.6) | 7(30.4) | 3(60.0) |  |
| Gender |  |  |  |  |  | 0.297 |
| Male | 68(71.6) | 31(64.6) | 14(73.7) | 18(78.3) | 5(100) |  |
| Female | 27(28.4) | 17(35.4) | 5(26.3) | 5(21.7) | 0 |  |
| Smoking status, n (%) |  |  |  |  |  | 0.647 |
| Never | 30(31.6) | 18(37.5) | 5(26.3) | 6(26.1) | 1(20.0) |  |
| Former/current | 65(68.4) | 30(62.5) | 14(73.7) | 17(73.9) | 4(80.0) |  |
| Stage,n(%) |  |  |  |  |  | 0.898 |
| IIIB-IIIC | 11(11.6) | 6(12.5) | 2(10.5) | 2(8.7) | 1(20.0) |  |
| IV | 84(88.4) | 42(87.5) | 17(89.5) | 21(91.3) | 4(80.0) |  |
| ECOG performance status,n(%) |  |  |  |  |  | 0.030  *Pa*=0.008 |
| 0-1 | 89(93.7) | 47(97.9)*^a^* | 15(79.0)*^a^* | 22(95.7) | 5(100) |  |
| 2 | 6(6.3) | 1(2.1) | 4(21.0) | 1(4.4) | 0 |  |
| PD-L1 TPS, n(%) |  |  |  |  |  | 0.317 |
| Unavailable | 74(77.9) | 35(72.9) | 18(94.7) | 18(78.3) | 3(60.0) |  |
| ≤1 % | 5(5.3) | 3(6.3) | 0 | 1(4.4) | 1(20.0) |  |
| 1-49% | 4(4.2) | 1(2.1) | 1(5.3) | 2(8.7) | 0 |  |
| ≥50% | 12(12.6) | 9(18.8) | 0 | 2(8.7) | 1(20.0) |  |
| Brain metastases, n (%) |  |  |  |  |  | 0.191 |
| No | 75(79.0) | 40(83.3) | 12(63.2) | 18(78.3) | 5(100) |  |
| Yes | 20(21.0) | 8(16.7) | 7(36.8) | 5(21.7) | 0 |  |
| Liver metastases, n(%) |  |  |  |  |  | 0.337 |
| No | 89(93.7) | 45(93.8) | 19(100) | 20(87.0) | 5(100) |  |
| Yes | 6(6.3) | 3(6.3) | 0 | 3(13.0) | 0 |  |
| Bone metastases, n(%) |  |  |  |  |  | 0.217 |
| No | 60(63.2) | 34(70.8) | 9(47.4) | 15(65.2) | 2(40.0) |  |
| Yes | 35(36.8) | 14(29.2) | 10(52.6) | 8(34.8) | 3(60.0) |  |
| Thoracic radiotherapy ,n(%) |  |  |  |  |  | 0.511 |
| No | 78(82.1) | 41(85.4) | 16(84.2) | 18(78.3) | 5(100) |  |
| Yes | 17(17.9) | 7(14.6) | 3(15.8) | 5(21.7) | 0 |  |
| Treatment strategy, n(%) |  |  |  |  |  | 0.572 |
| Monotherapy | 2(2.1) | 2(4.2) | 0 | 0 | 0 |  |
| Combination with chemotherapy | 93(97.9) | 46(95.8) | 19(100) | 23(100) | 5(100) |  |

Table S3. Demographic and clinical characteristics of the squamous NSCLC patients receiving second or later-line treatment with PD-1 inhibitors.

| Characteristics | Total  (N=59) | pembrolizumab  (N=10) | sintilimab  (N=20) | tislelizumab  (N=20) | camrelizumab  (N=9) | *P* value |
| --- | --- | --- | --- | --- | --- | --- |
| Median age(range),years | 64(48-79) | 64(54-79) | 66(48-79) | 63(52-76) | 60(53-67) | 0.265 |
| Age,years |  |  |  |  |  | 0.041  *Pb*=0.007 |
| ≤65 | 34(57.6) | 6(60.0) | 7(35.0)*^b^* | 13(65.0) | 8(88.9)*^b^* |  |
| ＞65 | 25(42.37) | 4(40.0) | 13(65.0) | 7(35.0) | 1(11.1) |  |
| Gender |  |  |  |  |  | 0.648 |
| Male | 53(89.8) | 10(100) | 17(85.0) | 18(90.0) | 8(88.9) |  |
| Female | 6(10.2) | 0 | 3(15.0) | 2(10.0) | 1(11.1) |  |
| Smoking status, n (%) |  |  |  |  |  | 0.455 |
| Never | 9(15.3) | 0 | 4(20.0) | 4(20.0) | 1(11.1) |  |
| Former/current | 50(84.7) | 10(100) | 16(80.0) | 16(8.0) | 8(88.9) |  |
| Stage,n(%) |  |  |  |  |  | 0.141 |
| IIIB-IIIC | 18(30.5) | 6(60.0) | 4(20.0) | 5(25.0) | 3(33.3) |  |
| IV | 41(69.5) | 4(40.0) | 16(80.0) | 15(75.0) | 6(66.7) |  |
| ECOG performance status,n(%) |  |  |  |  |  | 0.961 |
| 0-1 | 52(88.1) | 9(90.0) | 18(90.0) | 17(85.0) | 8(99.0) |  |
| 2 | 7(11.9) | 1(10.0) | 2(10.0) | 3(15.0) | 1(11.1) |  |
| PD-L1 TPS, n(%) |  |  |  |  |  | 0.146 |
| Unavailable | 43(72.9) | 6(60.0) | 18(90.0) | 15(75.0) | 4(44.4) |  |
| ≤1 % | 6(10.2) | 3(30.0) | 0 | 1(5.0) | 2(22.2) |  |
| 1-49% | 5(8.5) | 0 | 1(5.0) | 2(10.0) | 2(22.2) |  |
| ≥50% | 5(8.5) | 1(10.0) | 1(5.0) | 2(10.0) | 1(11.1) |  |
| Brain metastases, n (%) |  |  |  |  |  | 0.215 |
| No | 50(84.8) | 9(90.0) | 16(80.0) | 19(95.0) | 6(66.7) |  |
| Yes | 9(15.3) | 1(10.0) | 4(20.0) | 1(5.0) | 3(33.3) |  |
| Liver metastases, n(%) |  |  |  |  |  | 0.322 |
| No | 54(91.5) | 10(100) | 19(95.0) | 18(90.0) | 7(77.8) |  |
| Yes | 5(8.5) | 0 | 1(5.0) | 2(10.0) | 2(22.2) |  |
| Bone metastases, n(%) |  |  |  |  |  | 0.920 |
| No | 42(71.2) | 8(80.0) | 14(70.0) | 14(70.0) | 6(66.7) |  |
| Yes | 17(28.8) | 2(20.0) | 6(30.0) | 6(30.0) | 3(33.3) |  |
| Thoracic radiotherapy ,n(%) |  |  |  |  |  | 0.128 |
| No | 38(64.4) | 5(50.0) | 17(85.0) | 11(55.0) | 5(55.6) |  |
| Yes | 21(35.6) | 5(50.0) | 3(15.0) | 9(45.0) | 4(44.4) |  |
| Treatment strategy, n(%) |  |  |  |  |  | 0.473 |
| Monotherapy | 2(3.4) | 1(10.0) | 0 | 1(5.0) | 0 |  |
| Combination with chemotherapy | 57(96.6) | 9(90.0) | 20(100) | 19(95.0) | 9(100) |  |

Table S4. Demographic and clinical characteristics of the nonsquamous NSCLC patients receiving second or later-line treatment with PD-1 inhibitors.

| Characteristics | Total  (N=71) | pembrolizumab  (N=23) | sintilimab  (N=30) | tislelizumab  (N=8) | camrelizumab  (N=10) | *P* value |
| --- | --- | --- | --- | --- | --- | --- |
| Median age(range),years | 60(36-85) | 60(36-85) | 60(41-79) | 65(59-73) | 60(39-84) | 0.726 |
| Age,years |  |  |  |  |  | 0.730 |
| ≤65 | 45(63.4) | 14(60.9) | 21(70.0) | 4(50.0) | 6(60.0) |  |
| ＞65 | 26(36.6) | 9(39.1) | 9(30.0) | 4(50.0) | 4(40.0) |  |
| Gender |  |  |  |  |  | 0.844 |
| Male | 50(70.4) | 15(65.2) | 21(70.0) | 6(75.0) | 8(80.0) |  |
| Female | 21(29.6) | 8(34.8) | 9(30.0) | 2(25.0) | 2(20.0) |  |
| Smoking status, n (%) |  |  |  |  |  | 0.927 |
| Never | 24(33.8) | 8(34.8) | 11(36.7) | 2(25.0) | 3(30.0) |  |
| Former/current | 47(66.2) | 15(65.2) | 19(63.3) | 6(75.0) | 7(70.0) |  |
| Stage,n(%) |  |  |  |  |  | 0.028  *Pc*=0.002 |
| IIIB-IIIC | 9(12.8) | 3(13.0) | 1(3.3)*^c^* | 1(12.5) | 4(40.0)*^c^* |  |
| IV | 62(87.3) | 20(87.0) | 29(96.7) | 7(87.5) | 6(60.0) |  |
| ECOG performance status,n(%) |  |  |  |  |  | 0.358 |
| 0-1 | 64(90.1) | 22(95.7) | 25(83.3) | 8(100) | 9(90.0) |  |
| 2 | 7(9.9) | 1(4.4) | 5(16.7) | 0 | 1(10.0) |  |
| PD-L1 TPS, n(%) |  |  |  |  |  | 0.572 |
| Unavailable | 55(77.5) | 18(78.3) | 25(83.3) | 5(62.5) | 7(70.0) |  |
| ≤1 % | 3(4.2) | 1(4.4) | 1(3.3) | 1(12.5) | 0 |  |
| 1-49% | 7(9.9) | 2(8.7) | 3(10.0) | 0 | 2(20.0) |  |
| ≥50% | 6(8.5) | 2(8.7) | 1(3.3) | 2(25.0) | 1(10.0) |  |
| Brain metastases, n (%) |  |  |  |  |  | 0.326 |
| No | 45(63.4) | 17(79.3) | 19(63.3) | 3(37.5) | 6(60.0) |  |
| Yes | 26(36.6) | 6(26.1) | 11(36.7) | 5(62.5) | 4(40.0) |  |
| Liver metastases, n(%) |  |  |  |  |  | 0.796 |
| No | 61(85.9) | 21(91.3) | 25(83.3) | 7(87.5) | 8(80.0) |  |
| Yes | 10(14.1) | 2(8.7) | 5(16.7) | 1(12.5) | 2(20.0) |  |
| Bone metastases, n(%) |  |  |  |  |  | 0.983 |
| No | 46(64.8) | 15(65.2) | 20(66.7) | 5(62.5) | 6(60.0) |  |
| Yes | 25(35.2) | 8(34.8) | 10(33.3) | 3(37.5) | 4(40.0) |  |
| Thoracic radiotherapy ,n(%) |  |  |  |  |  | 0.134 |
| No | 56(78.9) | 21(91.3) | 21(70.0) | 5(62.5) | 9(90.0) |  |
| Yes | 15(21.1) | 2(8.7) | 9(30.0) | 3(37.5) | 1(10.0) |  |
| Treatment strategy, n(%) |  |  |  |  |  | 0.603 |
| Monotherapy | 3(4.2) | 1(4.4) | 1(3.3) | 1(12.5) | 0 |  |
| Combination with chemotherapy | 68(95.8) | 22(95.6) | 29(96.7) | 7(87.5) | 10(100) |  |

Table S5. Best objective response to treatment by RECIST 1.1 of squamous NSCLC patients after use of first-line immunotherapy.

| Best objective response | Pembrolizumab  (n=55) | Sintilimab  (n=46) | Tislelizumab  (n=72) | Camrelizumab  (n=11) | *P* value |
| --- | --- | --- | --- | --- | --- |
| CR,n(%) | 0 | 0 | 0 | 0 |  |
| PR,n(%) | 32(58.2) | 24(52.2) | 42(58.3) | 7(63.6) |  |
| SD,n(%) | 19(34.5) | 18(39.1) | 25(34.7) | 3(27.3) |  |
| PD,n(%) | 4(7.3) | 4(8.7) | 5(6.9) | 1(9.1) |  |
| ORR,% | 58.2 | 52.2 | 58.3 | 63.6 | 0.869 |
| DCR,% | 92.7 | 91.3 | 93.1 | 90.9 | 0.983 |

Abbreviations:

^a^ CR,complete response; PR,partial response; SD,stable disease; ORR,objective response; DCR,disease control rate

^b^ SD was defined per RECIST v1.1 as neither sufficient shrinkage to qualify for PR nor sufficient increase to qualify for PD (i.e., non-CR/non-PD)

Table S6. Best objective response to treatment by RECIST 1.1 of nonsquamous NSCLC patients after use of first-line immunotherapy.

| Best objective response | Pembrolizumab  (n=48) | Sintilimab  (n=19) | Tislelizumab  (n=23) | Camrelizumab  (n=5) | *P* value |
| --- | --- | --- | --- | --- | --- |
| CR,n(%) | 0 | 0 | 0 | 0 |  |
| PR,n(%) | 24(50.0) | 11(57.9) | 14(60.9) | 3(60.0) |  |
| SD,n(%) | 19(39.6) | 6(31.6) | 7(30.4) | 1(20.0) |  |
| PD,n(%) | 5(10.4) | 2(10.5) | 2(8.7) | 1(20.0) |  |
| ORR,% | 50.0 | 57.9 | 60.9 | 60.0 | 0.822 |
| DCR,% | 89.6 | 89.5 | 91.3 | 80.0 | 0.906 |

Table S7. Best objective response to treatment by RECIST 1.1 of squamous NSCLC patients after use of second or later-line immunotherapy.

| Best objective response | Pembrolizumab  (n=10) | Sintilimab  (n=20) | Tislelizumab  (n=20) | Camrelizumab  (n=9) | *P* value |
| --- | --- | --- | --- | --- | --- |
| CR,n(%) | 0 | 0 | 0 | 0 |  |
| PR,n(%) | 4(40.0) | 7(35.0) | 6(30.0) | 3(33.3) |  |
| SD,n(%) | 4(40.0) | 9(45.0) | 9(45.0) | 3(33.3) |  |
| PD,n(%) | 2(20.0) | 4(20.0) | 5(25.0) | 3(33.3) |  |
| ORR,% | 40.0 | 35.0 | 30.0 | 33.3 | 0.957 |
| DCR,% | 80.0 | 80.0 | 75.0 | 75.0 | 0.872 |

Table S8. Best objective response to treatment by RECIST 1.1 of nonsquamous NSCLC patients after use of second or later-line immunotherapy.

| Best objective response | Pembrolizumab  (n=23) | Sintilimab  (n=30) | Tislelizumab  (n=8) | Camrelizumab  (n=10) | *P* value |
| --- | --- | --- | --- | --- | --- |
| CR,n(%) | 0 | 0 | 0 | 0 |  |
| PR,n(%) | 10(43.5) | 11(36.7) | 3(37.5) | 3(30.0) |  |
| SD,n(%) | 10(43.5) | 16(53.3) | 4(50.0) | 5(50.0) |  |
| PD,n(%) | 3(13.0) | 3(10.0) | 1(12.5) | 2(20.0) |  |
| ORR,% | 43.5 | 36.7 | 37.5 | 30.0 | 0.899 |
| DCR,% | 87.0 | 90.0 | 87.5 | 80.0 | 0.877 |
